# Factors Associated with Carriage of Enteropathogenic and Non-Enteropathogenic Viruses: A Reanalysis of Matched Case-Control Data from the AFRIBIOTA Site in Antananarivo, Madagascar

**DOI:** 10.3390/pathogens12081009

**Published:** 2023-08-02

**Authors:** Iony Manitra Razanajatovo, Lova Andrianomiadana, Azimdine Habib, Mirella Malala Randrianarisoa, Helisoa Razafimanjato, Maheninasy Rakotondrainipiana, Prisca Andriantsalama, Ravaka Randriamparany, Soa Fy Andriamandimby, Pascale Vonaesch, Philippe Jean Sansonetti, Vincent Lacoste, Rindra Vatosoa Randremanana, Jean-Marc Collard, Jean-Michel Heraud, on behalf of the Afribiota Investigators

**Affiliations:** 1 Virology Unit, Institut Pasteur de Madagascar, Antananarivo 101, Madagascar; ionyr@pasteur.mg (I.M.R.); rhelisoa@pasteur.mg (H.R.); soafy@pasteur.mg (S.F.A.); vlacoste@pasteur.mg (V.L.); 2 Experimental Bacteriology Unit, Institut Pasteur de Madagascar, Antananarivo 101, Madagascarazimdine.habib@pasteur.fr (A.H.); jean-marc.collard@pasteur.fr (J.-M.C.); 3 Epidemiology and Clinical Research Unit, Institut Pasteur de Madagascar, Antananarivo 101, Madagascar; mirella@pasteur.mg (M.M.R.); r.maheninasy@gmail.com (M.R.); pandriatsalama@pasteur.mg (P.A.); rn.randriamparany@pasteur.mg (R.R.); rrandrem@pasteur.mg (R.V.R.); 4 Department of Fundamental Microbiology, University of Lausanne, 1002 Lausanne, Switzerland; pascale.vonaesch@unil.ch; 5 Unité de Pathogénie Microbienne Moléculaire, Institut Pasteur, 75015 Paris, France; 6 Chaire de Microbiologie et Maladies Infectieuses, Collège de France, 75000 Paris, France; philippe.sansonetti@pasteur.fr

**Keywords:** enteric viruses, stunting, Madagascar, Africa

## Abstract

Environmental Enteric Dysfunction (EED) is an associate driver of stunting in poor settings, and intestinal infections indirectly contribute to the pathophysiology underlying EED. Our work aimed at assessing whether enteric viral carriage is determinant to stunting. A total of 464 healthy and asymptomatic children, aged 2 to 5 years, were recruited, and classified as non-stunted, moderately stunted, or severely stunted. Among the recruited children, 329 stool samples were obtained and screened for enteric and non-enteric viruses by real-time polymerase chain reaction. We statistically tested for the associations between enteric viral and potential risk factors. Approximately 51.7% of the stool samples were positive for at least one virus and 40.7% were positive for non-enteric adenoviruses. No statistical difference was observed between virus prevalence and the growth status of the children. We did not find any statistically significant association between viral infection and most of the socio-demographic risk factors studied, except for having an inadequate food quality score or an over-nourished mother. In addition, being positive for *Ascaris lumbricoides* was identified as a protective factor against viral infection. In conclusion, we did not find evidence of a direct link between stunting and enteropathogenic viral carriage in our population.

## 1. Introduction

Stunting remains a major public health and economic development problem in Low- and Middle-Income Countries (LMICs). In 2021, it was estimated that four out of ten children suffered from stunting in Madagascar, one of the highest rates in the world. However, over the past decade, this prevalence has gradually decreased, from 50.1% in 2008–2009 to 39.8% in 2021 [1,2]. This gradual improvement can be explained by the programmatic approaches that have been implemented and the increased coordination of nutrition and health services through strong partnership between health authorities and several international agencies, such as the United Nations Children’s Fund (UNICEF) and the World Health Organization (WHO) [3]. Several factors are associated with stunting, and although a poor diet is one of the main causes of stunting, undernutrition is often exacerbated by disease and poor health [2]. Environmental Enteric Dysfunction (EED) may be an associate driver of stunting in LMICs [4,5]. EED is a chronic inflammatory condition of the gut that occurs in children living in unsanitary conditions, or among adults returning from deployment to LMICs. It is estimated that more than 75% of children in LMICs could be affected by, and suffer from, this syndrome at different degrees of severity [6]. In addition, intestinal infections (gastroenteritis), whether symptomatic or not [4,6,7], are suspected to result in abnormal gut microbiota and Small Intestinal Bacterial Overgrowth (SIBO) [8,9], both of which contribute to the pathophysiology underlying EED [6].

Many studies have examined the composition and function of the microbiome in the intestine [10,11,12,13,14,15]. Some have suggested that the virome component of the intestinal microbiota provides protection against pathologic intestinal inflammation, similar to the well-established protective role of commensal bacteria [16]. However, some pathogenic viruses can induce gut inflammation, including non-bacterial gastroenteritis and diarrhea [7]. The spectrum of symptoms can range from an asymptomatic infection to severe diseases with dehydration. Four viral families are commonly associated with gastroenteritis: *Caliciviridae* (including norovirus (NoV) and Sapovirus), *Reoviridae* (rotaviruses (RoV)), *Astroviridae* (astroviruses (AstV)), and *Adenoviridae* (adenoviruses (AdV)). Adenoviruses, especially types 40 and 41, are second to rotaviruses as a cause of acute diarrhea in young children according to two recent studies of diarrheal etiology in LMICs [17,18,19]. The infection is spread by fecal–oral transmission and in respiratory droplets of moisture, such as those produced by coughing [20], and adenoviruses may be excreted in stools for prolonged periods of time by young children with no evidence of disease [21]. In addition, NoV can be found in the respiratory tract and in stool samples of children with and without acute gastroenteritis symptoms [20]. In Madagascar, a previous study has shown that RoV were detected in 6.2% of children ≤5 years with diarrhea [22]. Moreover, NoV and AstV were detected in 6.0% and 2.1% of children under 16 years presenting with acute gastroenteritis, respectively [23,24]. A case–control study with children aged 0 to 59 months conducted between 2011 and 2014 in urban and semi-urban areas found that RoV were the main pathogens detected (43.4%) and that they were strongly associated with severe diarrhea [25].

In the framework of the AFRIBIOTA (AFRIcan MicroBIOTA) project, a case–control study for stunting was conducted in Antananarivo (capital of Madagascar) and Bangui (capital of the Central African Republic), in children aged 2–5 years with no overt signs of gastrointestinal disease [10]. Previous research under AFRIBIOTA found a high proportion of enteropathogens in both stunted and non-stunted children, suggesting that carriage of enteropathogens alone could not be directly associated with stunting [6,11,12,13]. Furthermore, it was demonstrated that intestinal helminthic and protozoan infections were widespread, yet not associated with stunting in the group of children studied [13]. While the role of viruses in EED has been addressed in different settings [14,15,16,17], it had not been studied in the group of children from Madagascar. Our study aimed at identifying factors associated with viral carriage of rotaviruses, Norovirus GII, astroviruses or adenoviruses according to nutritional status in the study population recruited in Antananarivo, Madagascar.

## 2. Materials and Methods

### 2.1. Study Design/Recruitment of Participants

This case control study was extensively described in a previous publication [10]. Briefly, the study population restricted to Antananarivo, Madagascar, comprises HIV-negative children aged 2 to 5 years, neither suffering from acute malnutrition, nor from any other severe disease (such as dysenteric syndrome, severe acute respiratory infection (SARI)/influenza-like illness (ILI), meningitis, malaria, acute otitis media, varicella, measles). Children were recruited in the capital city, in two neighborhoods (Ankasina and Andranomanalina Isotry), and in two health centers (Centre Hospitalier Universitaire Joseph Ravoahangy Andrianavalona—CHUJRA and Centre de Santé Materno-Infantile de Tsaralalana—CSMI). Children were recruited either in the community (community-recruited children) or directly at the hospital (hospital-recruited children). After HIV testing, to exclude seropositive children, we enrolled a total of 464 children. Trained health professionals conducted sampling at health centers.

### 2.2. Data Collection

To assess risk factors for acquiring pathogens, a questionnaire was administered to children and their caregivers, and the collected subset of data were analyzed. In brief, the questionnaire contained four sections: 1. Socio-demographic data: age, gender, community setting, education of the mother; 2. Environmental factors: housing conditions and quality of drinking water; 3. Behavior habits: hand washing, foods consumption, and exposure to sewage and garbage; 4. Medical status/history: fever, diarrhea, rotavirus vaccination, parasitic carriage, stuffy nose, rhinorrhea, and cough [10]. Foods consumed by children the day before the survey were classified into seven food groups according to WHO classification: (1) grains, roots and tubers; (2) legumes and nuts; (3) dairy products; (4) flesh foods (meats/fish/poultry); (5) eggs; (6) fruits and vegetables; and (7) oils and fats. The Dietary Diversity Score (DDS) is calculated based on the number of food groups consumed by the child 24 h before the survey. A Dietary Diversity Score (DDS) of four is considered the minimum DDS for adequate food diversity. Accordingly, a child with a DDS *<* 4 was classified as having unsuitable food diversity score; otherwise, they were considered to have adequate dietary diversity [26]. In addition, we also collected anthropometric measurements (height and weight) as previously described. The children were classified according to the median height of the WHO reference population [27] in three different groups: severe stunting (SS) (height-for-age z-score ≤ −3 SD), moderate stunting (MS) (height-for-age z-score between −3 SD and −2 SD) and non-stunted (NS) (height-for-age z-score ≥ −2 SD). The NS individuals were matched for living area and sampling time-period and were recruited during the entire study period (December 2016–March 2018). 

### 2.3. Sample Collection

The collection of stool samples has previously been described in Vonaesch et al., 2018 [10]. Stools (around 10 g) were collected in the morning at the hospital (directly before coming to the hospital for the community recruited children) and the time of defecation recorded. If community-recruited children were able to emit again feces at the hospital, these feces were also collected. All stools were collected in appropriate container then dispatched into cryotubes and directly snap-frozen in liquid nitrogen before being shipped to the Institut Pasteur de Madagascar and transferred into −80 °C freezer until laboratory experiment processing.

### 2.4. Extraction of Nucleic Acids

DNA and RNA extraction methods have previously been described by Collard et al., 2022 [6]. Briefly, RNA and DNA were extracted by commercial kits using a Qiacube instrument (Cador Pathogen 96 QIAcube HT Kit, Qiagen France SAS, Courtaboeuf, France), and following the manufacturer’s recommendations with an additional bead-beating step to increase mechanical disruption. Freshly thawed 200 mg sample were mixed with ASL buffer at 4 °C and vigorously vortexed for 1 min. The suspension was transferred into a Pathogen Lysis Tube (Qiagen) containing two mg of sterile glass beads (100 μM diameter) and disrupted mechanically using a TissueLyser II (Qiagen GmbH, Hilden, Germany) for 10 min at 30 Hz. The suspension was then incubated at 95 °C for 5 min, vortexed for 15 s and centrifuged at 14,000 rpm for 1 min to eliminate any solid particles in subsequent steps. All samples were eluted in 150 μL AE buffer. Concentrations and purity of RNA and DNA were assessed by spectrophotometry (Nanodrop 2000 Spectrophotometer, Thermo Fisher Scientific, Waltham, MA, USA) via 260/280 and 260/230 absorbance ratios. Nucleic acids extracts were stored at −80 °C until further analyses.

### 2.5. Real-Time PCR and RT-PCR (qPCR and RTqPCR)

The primers and probes used for real-time RT-PCR or PCR are listed in Supplementary Table S1. The targeted regions of rotavirus, norovirus GII, astrovirus, pan-adenovirus, and adenovirus genogroup types F40/41 are located in conserved regions of their genomes [27]. Amplifications were carried out in a ThermoFischer QuantStudio 5 instrument (Applied Biosystems, Port-Louis, Mauritius). Real-time PCR amplification reactions were performed in a single run using the Superscript^®^III Platinum Taq DNA polymerase One-Step qRT-PCR (Invitrogen, Carlsbad, CA, USA) and the described primers [27] as follows: reverse transcription of RNA was performed at 50 °C for 15 min, followed by 45 cycles of a two-step PCR (95 °C for 15 s and 60 °C for 30 s). The results were recorded as the Ct value, which is inversely related to the viral load in each specimen. A specimen was considered positive if the Ct value was ≤39. Standard curves for each target were established. Negative and positive controls were included in each run. The efficacy of amplification was assessed for each real-time PCR by analyzing serial dilutions of pUC57 plasmids carrying all synthetic target inserts (GeneCust Europe, Dudelange, Luxembourg).

The molecular screening of parasitic carriage was described in a previous study [13], and the resulting datasets were analyzed in this study. The investigated parasites were *Giardia intestinalis*, *Ascaris lumbricoides*, *Trichuris trichiura* and *Enterobius vermicularis*.

### 2.6. Statistical Analysis

The statistical analysis was performed with R-Studio (version 4.0.4; The R Foundation for Statistical Computing, Vienna, Austria). We assessed the associations between enteric viral infection (positivity for any of the four enteropathogenic viruses’ groups: rotaviruses, norovirus GII, astroviruses and adenoviruses—not counting non-enteric adenovirus) and potential risk factors (exposure variables). Fisher’s exact test/Chi-square test, or Student test/Wilcoxon test (according to their conditions of use) were performed to analyze a statistically significant difference (*p* ≤ 0.05). We then checked the associations between exposure variables. Categorical variables were expressed as percentages; quantitative variables were expressed as a mean (+/− Standard Deviation) or median (interquartile range). The nutritional statuses, stunted vs. non-stunted, were compared using Chi-square test or Fisher Exact test for qualitative variables, and the Student t test or the Mann–Whitney U test was used for quantitative variables. We used a multivariable logistic regression model to identify independent predictors of viral infection using a manual backward selection approach. All exposure variables with a *p*-value ≤ 0.2 in the univariable or variables considered as potential confounding factors were assessed in the initial model, and the likelihood ratio test was used at each step of the selection model. Exposure variables were retained in the model if the test was statistically significant (*p*-value ≤ 0.05). The variables age, gender and season of inclusion were used to match case and control. As we did not get a perfect matching, we forced these variables in the multivariate model. Confounding factors were checked in the final model. We then assessed the goodness of fit of the final model using tests of model adequacy (Hosmere and Lemeshow test or Pearson Chi-squared) and using the ROC curve using the AUC (area under curve). The threshold of 0.5 to calculate the sensitivity and specificity of the model was considered.

## 3. Results

### 3.1. Description of the Study Population

**Figure 1:**
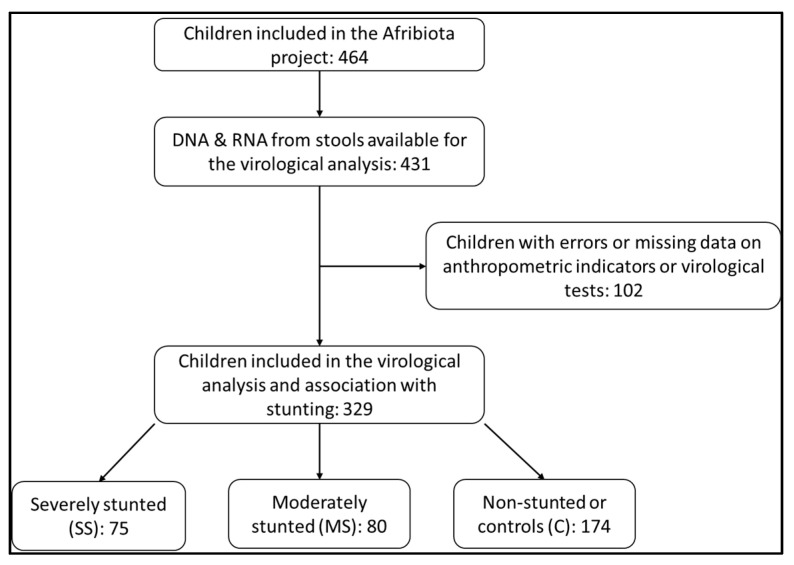
Flowchart of the inclusion process of HIV-negative children aged 2 to 5 years, recruited in Ankasina or Andranomanalina Isotry, Antananarivo, Madagascar, from December 2016 to March 2018.

During the entire period of the AFRIBIOTA project, we enrolled 464 children between 24 and 59 months of age, of whom 329 provided enough stool for nucleic acid extraction and virological analyses. Of the 329 children, 75 were severely stunted (SS), 80 moderately stunted (MS) and 174 non-stunted (NS) (Figure 1). Their age ranged from 33 to 53 months and the sex ratio (M/F) was 0.91.

### 3.2. Detection of Viruses in the Study Population

**Figure 2:**
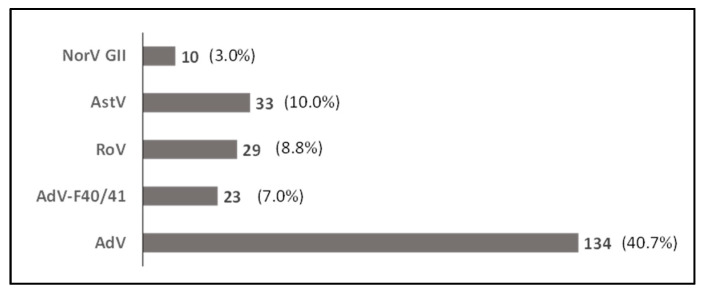
Prevalence of viruses detected in stool samples of the study population; all nutritional status considered (N = 329). AdV: Adenovirus; AdV-F40/41: Adenovirus type F40 and F41; RoV: Rotavirus; AstV: Astrovirus; NorV GII: Norovirus GII.

Of the 329 stools tested, 170 (51.7%) were positive for at least one of the targeted viruses. Among the positives, 142 (83.5%) were infected with one virus, 20 (11.8%) with two viruses and 8 (4.7%) with three viruses (Supplementary Table S2). AdVs were the most prevalent viruses detected, with 134 (40.7%) positive stools, followed by AstV, RoV, and NoV, with, respectively, 33 (10.0%), 29 (8.8%) and 10 (3.0%) of positive cases (Figure 2). Adenovirus type F40/41 was detected in 23 (7.0%) stools. Interestingly, most of the RoVs (72.4%; 21/29) were detected as co-infection with one or two other viruses.

**Figure 3:**
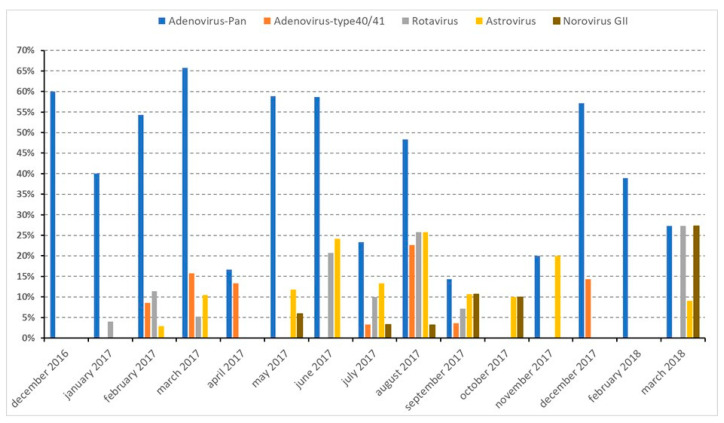
Prevalence of viral infections during the study period (December 2016–March 2018).

No statistical differences were observed between the prevalence of viruses targeted and the growth status of children (Table 1). Although not significant, we noted that the positivity rates of all viruses tested were higher among NS children. Looking at their distribution over time, at least one of the four types of viruses was detected each month during the study period (Figure 3). Adenoviruses were detected monthly, from December 2016 through March 2018, except in October 2017, while adenovirus type F40/41, norovirus GII, astrovirus and rotavirus were detected more sporadically.

To determine whether there was an association between the nutritional status of children and being infected with adenovirus type F40/41, we restricted the analysis to the periods when these viruses were detected (February–April 2017; July–September 2017; December 2017). No statistically significant association was observed (*p* = 0.72) (Supplementary Table S2).

### 3.3. Characteristics of the Study Population Associated with Viral Infection

By examining the socio-demographic data of the population studied, no statistical differences were observed regarding viral infection for the sex, age, and weight at birth (Table 2). In addition, no statistically significant differences were observed for the following variables: growth status, chronic malnutrition, history of acute malnutrition, diarrhea, respiratory infection, fever, rotavirus vaccination and parasitic carriage. The only piece of socio-demographic evidence showing a statistically significant difference was the food diversity score (*p* = 0.035), with 58.9% of the children with an inadequate food diversity score being positive for a virus compared to 47% of those with an acceptable feeding. Our data showed that a viral infection was detected in 56% of severely stunted and in 52.9% of non-stunted children, while 45% of MS cases were positive for at least one virus. Furthermore, at the time of the study, almost all children had no symptoms of diarrhea (97.3%; 320/329), no history of diarrhea (96.1%; 316/329) nor any history of respiratory infection (97.3%; 320/329), but 66.3% had a history of fever and the rates of viral infection in each case were circa 50% (Table 2). At last, the rotavirus vaccination status of the children showed that 95.4% (312/329) were vaccinated and 52.2% of those who received at least one dose of vaccine were positive for viral infection, including rotavirus. Some children displayed clinical signs of respiratory infection, such as rhinorrhea (62.6%), stuffy nose (51.4%) and cough (36.8%). Association of these symptoms with viral infection showed no significant differences, even though children with stuffy nose, rhinorrhea and cough were positive to viral carriage in 50.3%, 49% and 47.9% of the cases, respectively.

Given that non-enteropathogenic adenoviruses were the most prevalent in each group of children, we assessed their possible association with these clinical signs by excluding the enteric virus type F40/41 carriage as well as co-infections with other enteric viruses. Non-enteric adenovirus infection alone was not statistically correlated with the age of the children, their growth status, nor with clinical signs such as stuffy nose, rhinorrhea, or cough (Supplementary Table S3). Among children displaying clinical signs, non-enteric adenoviruses were detected in 31.7% of those with stuffy nose (*p* = 0.111), 32.2% of those with rhinorrhea (*p* = 0.066) and 30.0% of those with cough (*p* = 0.102). For each symptom, these rates were lower than those obtained for children without clinical signs.

### 3.4. Characteristics of the Household Environment of the Study Population and Viral Infection

We observed that a higher proportion of children of over-nourished mothers (67.6%) were infected with a virus as compared to children of normal (45.9%) or undernourished (48.9%) mothers (Table 2). This difference was statistically significant with a *p*-value = 0.006. None of the other parameters considered showed a statistically significant difference.

At last, looking at the association of viral infection with parasitic infestation, we observed that 75.4% (248/329) of the study population was positive for at least one parasite, and 50% (124/248) of those positive for a parasite were also infected with a virus (Table 3). About a quarter (24.6%) of the children were positive for *Giardia intestinalis*, 49.2% for *Ascaris lumbricoides*, 65.6% for *Trichuris trichiura* and 0.9% for *Enterobius vermicularis*. Among those positive for one of these parasites, between 33.3% (*E. vermicularis*) and 52.3% (*T. trichiura*) were carrying a virus. A statistically significant difference was only detected for *A. lumbricoides* with a proportion of viral infection higher (58.3%) in “Ascaris-free” children as compared to infected ones (44.4%) (Table 3).

### 3.5. Risk Factors and Protective Factors Associated with Viral Infection

A bivariate analysis of risk factors associated with viral infection was carried out. Then, a multivariate analysis of resulting predictor variables with *p*-value ≤ 0.20 was undertaken to determine any collinearity (Supplementary Tables S4 and S5). The identified predictor variables were food score diversity of the child, nutritional status of the mother, weight at birth, age, sex, respiratory infection history, drinking water treatment, *A. lumbricoides* infestation, whether the child ate in the plate of the guardian, mother’s schooling level and household waste disposal. An association was observed between the food diversity score and drinking water treatment (*p* < 0.001), as well as between the food diversity score and mother’s schooling level (*p* = 0.003). In addition, mother’s schooling level was associated with *A. lumbricoides* carriage (*p* = 0.017).

We observed that children with an adequate food diversity score were less likely (ORaj = 0.59, CI 95% (0.37–0.95)) to be infected with viruses than those with an inadequate food diversity score, controlling for the effects of mother nutritional status and *A. lumbricoides* infestation (Table 4). Children without *A. lumbricoides* infestation were more likely to have a viral infection than those infested by this type of parasite, controlling for the effect of children’s food diversity score and mother nutritional status (ORaj = 1.79, CI 95% (1.12–2.86)). Moreover, children whose mother was overweighted were more likely (OR = 2.37, CI 95% (1.33–4.21)) to be infected with viruses than those with a mother having a normal weight or being underweighted, controlling for the effect of children’s diversity score and *A. lumbricoides* infestation. The *p*-values of the Hosmer–Lemeshow and Pearson residual tests were, respectively, <2.2 × 10^−16^ and 0.383, indicating an insufficient model fit of the data.

## 4. Discussion

Stunting is major public health and development concern for LMICs such as Madagascar. Stunting syndrome or linear growth delay, linked to poor nutrition and repeated infections such as intestinal infections, indirectly contribute to the pathophysiology underlying EED [4,5]. As a matter of fact, stunting has a permanent impact on the physical growth and intellectual development of children [1,5,8]. In recent years, a broad number of studies have addressed the implication of EED on children’s development [5,8,28,29,30]. Through the AFRIBIOTA project, pediatric environmental enteropathy was investigated, and the status of bacteria and parasites in the gut microbiota, as well as Small Intestinal Bacterial Overgrowth (SIBO), was evaluated in apparently healthy and stunted children from Antananarivo, Madagascar [6,11,12,13]. Although overgrowth of bacterial enteropathogens was detected at a higher rate in the duodenal fluid of asymptomatic stunted children [6], the current view of stunting associated with the overstimulation of the small intestine through recurrent infections is now challenged by the finding that oropharyngeal bacteria were also detected in the feces along the gastrointestinal microbiome through decompartmentalization [11]. A separate study on parasitic infections showed that the prevalence of intestinal parasites was substantial in both stunted and control children. No apparent differences were seen in stunted and healthy children [13]. 

In this study, we screened a range of viruses known to affect intestinal structure and function, such as enteric and non-enteric adenoviruses, rotaviruses, astroviruses and norovirus GII, for their presence and possible association with growth stunting in controlled and stunted children living in two districts of Antananarivo. Different parameters, ranging from living conditions, hygiene, and sanitation, as well as co-infestation with parasites, were evaluated to determine if they could correspond to risk factors or protective factors associated with viral carriage. Surprisingly, no significant differences were found, apart from children with an inadequate food quality score and of those of over-nourished mothers, who had a higher rate of viral infection than children with an adequate food quality score and normal or undernourished mothers. Variables commonly thought to be risk factors for enteropathies (e.g., WASH) showed no association with viral carriage. This lack of association might be explained firstly by random variation and secondly because enteric viruses might be less sensitive to hygiene conditions and individual characteristics in comparison with bacterial enteropathogens. In fact, in our previous studies, we found more consistent associations with the carriage of enteric bacteria [11] and the occurrence of stunting [12]. Given the number of associations tested, most of the variables commonly associated with growth delay and risk factors to enteropathy did not give statistically significant outcomes compared to a small number that came out as statistically significant at the α < 0.05, which is puzzling. We assumed that our analysis is underpowered and thus any possible explanation for the association found in children with an inadequate food quality score and those with over-nourished mothers could be due to random variation.

When considering a separate study of the Afribiota project that addresses the main analysis of risk factors for stunting [12], no association was found between food diversity and stunting, and higher education of the mother was found to be a protective factor for stunting. However, the most common stunting-associated factors globally cited in the literature were also reflected in the study, such as low birth weight, lack of access to soap, poor sanitation as well as comorbidities such as anemia and infections (diarrhea, dermatitis, or respiratory disease), which are risk factors. Additionally, it is believed that enteropathogens were considered the main taxa driving inflammation in undernutrition. In a parallel study, under the Afribiota project addressing bacterial composition of the microbiota in the gut of stunted children [11], it was observed that, due to microbial decompartmentalization of the oropharyngeal to gastrointestinal tract, there is an overrepresentation of enteropathogenic bacteria in the duodenum of stunted children compared to duodenal, gastric and stool samples of non-stunted or normal children. In sum, the profile of bacterial and viral infections or carriage in the same population cannot be compared, as in our case half of the children of the study population were infected with at least one virus at the time of inclusion.

Although this study has been undertaken with children recruited from two of the poorest neighborhoods in Antananarivo, it is worthwhile to note that previous studies in other countries have reported that higher rates of enteric viruses were also found in children of a higher socio-economic level whose hygienic behavior contributes to viral contamination [31]. In that case, young children are more exposed to diarrheal pathogens, by ingestion of contaminated material by aerosolized infectious particles from infected individuals or through fecal contamination of food, water, soil, and surfaces [9,32,33].

As a matter of fact, the gastrointestinal tract is a common site of infection by pathogenic viruses, bacteria, protozoa and helminths. Signals derived from commensal bacteria and helminth parasites can influence the mammalian immune response, and helminths are known to be able modulate the immune system [34]. We found it interesting to evaluate the association of parasites to viral carriage. The large roundworm *A. lumbricoides* is a common soil-transmitted helminth in developing countries [35]. *Ascaris* infestation is one of the most common human parasitic infestations worldwide, which causes approximately 60,000 deaths per year, mainly in children experiencing malnutrition and developmental deficits from chronic infection. Recent studies on virus-helminth co-infection have shown that *Ascaris* infestation in mammals can compromises the host cell control of other infectious agents, including *Mycobacterium tuberculosis*, *Plasmodium* spp., and HIV as well as responses to non-parasite antigens, thus hampering vaccination efficacy against other pathogens [34,35,36]. Unexpectedly, our findings show that, among our cohort, being infested with *A. lumbricoides* had a protective effect on viral carriage. These results demonstrate the complexity of interaction between virus and helminth within the host in real life. Several factors may play a role, including (i) the type of helminth and virus, (ii) the tissue tropism, (iii) the nature of the antiviral immune response and (iv) the timing of viral infection and the helminth life cycle [35].

The association of enteric viruses with diarrhea has been well established in symptomatic individuals. Apart from the classic enteropathogenic viruses, non-enteric (respiratory and keratoconjunctivitis) adenoviruses are known to replicate in the gut and to persist in a latent state following primary infection, most commonly in early childhood [33]. The shedding of non-enteric adenoviruses into the stool from asymptomatic individuals has been described [14,37] and suggested as a potential etiology of gastroenteritis in recent studies with hospitalized pediatric patients in Bangladesh, Thailand, and Italy [37,38,39]. In this study, we found that 40.7% of children were positive for adenoviruses (through a Pan-AdV PCR) and 7.0% for adenoviruses type F40/41. Our results show that enteropathogens (adenoviruses type F40/41, astrovirus, norovirus GII, rotavirus) were sporadically detected during the period of enrolment (December 2016–March 2018), while non-enteric adenoviruses were detected all over the study period. So far, Madagascar has no available data on the epidemiology of diarrheal diseases to evaluate the circulation of those pathogens. However, non-enteric adenoviruses were detected all year round in a previous study conducted on children under 5 years of age suffering from influenza-like illness in Madagascar [40]. Here, we observed the same pattern of circulation.

This study has some limitations. Norovirus GI and specifically Sapovirus testing were not included in this screening. Sapovirus testing could not be performed because of technical constraints, whereas in a previous study on diarrheic children of different age groups in Antananarivo, the circulation of norovirus GI had been barely detected [23]. Moreover, given that the panel of viral pathogens screened was not exhaustive, we cannot rule out the presence of other enteric and non-enteric viruses in our study population [14,15]. Previous studies conducted in Madagascar have demonstrated that non-polio enteroviruses were identified in 26.8% of stools collected from healthy children under five [41]. We may have thus underestimated the prevalence of enteric viruses in our population. Nevertheless, to limit this bias, we have targeted the main viral families associated with diarrheal diseases based on the literature and on previous Malagasy studies. 

Even if no association was found between stunting and viral carriage, our results reported that more than half of the children were infected with viruses and co-infections with viruses and bacteria are common. These data can serve as a baseline for public health interventions and community health programs. As suggested in previous separate studies on factors associated to stunting, including analysis of bacterial and parasitic enteropathogens, strategies such as water, sanitation, and hygiene (WASH) interventions should be strengthened [12,13] to reduce virus transmission through sensitization or the implementation of different activities. Moreover, programs addressing the improvement of a child’s diet such as a nutrition education program or interventions favoring food accessibility should be prioritized, as suggested in a previous study addressing the nutritional status of children in two distant districts of Madagascar, Moramanga (Middle East) and Morondava (South-West), where a low food diversity score was frequent, as more than one third of children (39.2%) had an inadequate food diversity score [42].

## 5. Conclusions

The stunted and controlled children recruited in this study were apparently healthy and not suffering from diarrhea, although some presented respiratory clinical manifestations such as stuffy nose, rhinorrhea, and cough. We did not find any evidence that enteropathogenic viral carriage had a direct link with stunting in our study population. Our analysis shows that respiratory clinical manifestations were not correlated with adenovirus infection, suggesting other causes. For example, it is known that enteric viruses, such as norovirus and rotavirus, can be detected in different sites, such as the lower airway tract beside the intestine replication sites, causing many upper respiratory symptoms, such as cough or runny nose. It is also possible that the children harbored other respiratory viruses or unknown viral infections not tested in this study.

## Figures and Tables

**Table 1:** Prevalence of viruses detected in stool samples from children according to their growth status (non-stunted, moderately and severely stunted).

Virus ^1^	NS ^2^ N_1_ (%)	MS ^2^ N_2_ (%)	SS ^2^ N_3_ (%)	Total N = 329	*p*-Value ^3^
AdV	75 (43.1)	27 (33.7)	32 (42.7)	134 (40.7)	0.344
AdV type F40/41	15 (8.6)	4 (5)	4 (5.3)	23 (7)	0.551
AstV	14 (8)	10 (12.5)	9 (12)	33 (10)	0.444
RoV	14 (8)	4 (5)	11 (14.7)	29 (8.8)	0.115
NorV GII	5 (2.9)	1 (1.25)	4 (5.3)	10 (3)	0.350

^1^ AdV: Adenovirus; AdV: Adenovirus; AdV-F40/41: Adenovirus type F40 and F41; RoV: Rotavirus; AstV: Astrovirus; NorV GII: Norovirus GII. ^2^ Growth status: NS = Non-Stunted (controls; N_1_ = 174); MS = Moderately Stunted (N_2_ = 80); SS = Severely Stunted (N_3_ = 75). ^3^ Comparisons between controls and stunted children (MS + SS) were determined using Pearson’s χ^2^-test or Fisher’s exact test according to their conditions of use. A *p*-value < 0.05 was considered statistically significant.

**Table 2:** Characteristics of the study population according to viral infection detected (N = 329).

Variable	Viral Detection		Total (N = 329)	*p*-Value
	No (N = 159)	Yes (N = 170)		
	n (%)/Median [IQR]	n (%)/Median [IQR]	n/Median [IQR]	
**Sex**				0.362 ^1^
Female	79 (45.9%)	93 (54.1%)	172	
Male	80 (51.0%)	77 (49.0%)	157	
**Age (months)**	44.28 [34.8–53.1]	42.131 [33.0–52.2]	43.232 [33.7–53.0]	0.256 ^2^
**Weight at birth**				0.085 ^3^
Mean (SD)	3.167 (0.668)	3.356 (0.681)	3.269 (0.680)	
Standard deviation	1.750–5.000	1.900–4.900	1.750–5.000	
**Food diversity score (child)**				0.035 ^1^
Inadequacy/insufficiency	53 (41.1%)	76 (58.9%)	129	
Acceptable	106 (53.0%)	94 (47.0%)	200	
**Growth status**				0.352 ^1^
Moderately stunted	44 (55.0%)	36 (45.0%)	80	
Severely stunted	33 (44.0%)	42 (56.0%)	75	
Non-stunted	82 (47.1%)	92 (52.9%)	174	
**Chronic malnutrition**				0.941 ^1^
Moderate chronic malnutrition	47 (49.5%)	48 (50.5%)	95	
Acute chronic malnutrition	37 (46.8%)	42 (53.2%)	79	
Normal nutrition	75 (48.4%)	80 (51.6%)	155	
**History of acute malnutrition**				1.000 ^4^
Yes	5 (50.0%)	5 (50.0%)	10	
No	154 (48.3%)	165 (51.7%)	319	
**Diarrhea**				0.323 ^4^
Yes	6 (66.7%)	3 (33.3%)	9	
No	153 (47.8%)	167 (52.2%)	320	
**History of diarrhea**				0.685 ^1^
Yes	7 (53.8%)	6 (46.2%)	13	
No	152 (48.1%)	164 (51.9%)	316	
**Respiratory infection history**				0.176 ^4^
Yes	2 (22.2%)	7 (77.8%)	9	
No	157 (49.1%)	163 (50.9%)	320	
**History of fever**				0.389 ^1^
Yes	101 (46.3%)	117 (53.7%)	218	
No	56 (51.4%)	53 (48.6%)	109	
**Rotavirus vaccination**				0.354 ^1^
At least one dose	149 (47.8%)	163 (52.2%)	312	
Zero dose	9 (60.0%)	6 (40.0%)	15	
**Stuffy nose**				0.608 ^1^
Yes	84 (49.7%)	85 (50.3%)	169	
No	75 (46.9%)	85 (53.1%)	160	
**Rhinorrhea**				0.214^1^
Yes	105 (51.0%)	101 (49.0%)	206	
No	54 (43.9%)	69 (56.1%)	123	
**Cough**				0.301 ^1^
Yes	63 (52.1%)	58 (47.9%)	121	
No	96 (46.2%)	112 (53.8%)	208	
**Number of children under 5-years old in the household**	1.0 [1.0–2.0]	1.0 [1.0–2.0]	1.0 [1.0–2.0]	0.627 ^1^
**Nutritional status of the mother**				0.006 ^2^
Normal	106 (54.1%)	90 (45.9%)	196	
Undernourished	24 (51.1%)	23 (48.9%)	47	
Overnourished	24 (32.4%)	50 (67.6%)	74	
**Education level of the mother**				0.258 ^3^
No schooling	3 (33.3%)	6 (66.7%)	9	
Elementary school	84 (53.8%)	72 (46.2%)	156	
Secondary School	58 (43.6%)	75 (56.4%)	133	
High School or above	12 (44.4%)	15 (55.6%)	27	
**Weaning age**				0.388 ^2^
≤12 months	19 (52.8%)	17 (47.2%)	36	
12–24 months	28 (41.2%)	40 (58.8%)	68	
≥24 months	97 (50.0%)	97 (50.0%)	194	
**Age at the introduction of first solid foods**				0.458 ^2^
<6 months	43 (45.3%)	52 (54.7%)	95	
≥6 months	115 (49.8%)	116 (50.2%)	231	
**Running water in the house**				0.695 ^2^
Yes	21 (45.7%)	25 (54.3%)	46	
No	138 (48.8%)	145 (51.2%)	283	
**Water used for child hygiene**				1.000 ^3^
Running water and other water sources	4 (50.0%)	4 (50.0%)	8	
Running water only	155 (48.4%)	165 (51.6%)	320	
**Water used for the child during the last 2 weeks**				1.000 ^3^
Running water	157 (48.6%)	166 (51.4%)	323	
Other water sources	2 (40.0%)	3 (60.0%)	5	
**Drinking water treatment**				0.107 ^2^
No	109 (45.6%)	130 (54.4%)	239	
Yes	50 (55.6%)	40 (44.4%)	90	
**Using soap in the household**				0.674 ^3^
Yes	83 (50.3%)	82 (49.7%)	165	
Sometimes	73 (46.8%)	83 (53.2%)	156	
No	3 (37.5%)	5 (62.5%)	8	
**Washing the hands of the child**				0.527 ^3^
No hand washing	0 (0.0%)	2 (100.0%)	2	
Only with cold/hot water	53 (48.2%)	57 (51.8%)	110	
Sometimes with water and soap	90 (50.3%)	89 (49.7%)	179	
Always with water and soap	16 (42.1%)	22 (57.9%)	38	
**Water used for child hygiene**				1.000 ^3^
Running water and other water sources	4 (50.0%)	4 (50.0%)	8	
Running water only	155 (48.4%)	165 (51.6%)	320	
**Water used for the child during the last 2 weeks**				1.000 ^3^
Running water	157 (48.6%)	166 (51.4%)	323	
Other water sources	2 (40.0%)	3 (60.0%)	5	
**Drinking water treatment**				0.107 ^2^
No	109 (45.6%)	130 (54.4%)	239	
Yes	50 (55.6%)	40 (44.4%)	90	
**Using soap in the household**				0.674 ^3^
Yes	83 (50.3%)	82 (49.7%)	165	
Sometimes	73 (46.8%)	83 (53.2%)	156	
No	3 (37.5%)	5 (62.5%)	8	
**Washing the hands of the child**				0.527 ^3^
No hand washing	0 (0.0%)	2 (100.0%)	2	
Only with cold/hot water	53 (48.2%)	57 (51.8%)	110	
Sometimes with water and soap	90 (50.3%)	89 (49.7%)	179	
Always with water and soap	16 (42.1%)	22 (57.9%)	38	
**Guardian’s hand washing**				1.000 ^3^
No hand washing	2 (40.0%)	3 (60.0%)	5	
Only with cold and/or hot water	92 (48.7%)	97 (51.3%)	189	
With water and soap	64 (48.5%)	68 (51.5%)	132	
**Number of handwashing of the guardian per day**				0.770 ^2^
1–2 times	12 (42.9%)	16 (57.1%)	28	
3–4 times	73 (50.0%)	73 (50.0%)	146	
≥5 times	73 (47.7%)	80 (52.3%)	153	
**Type of kitchen**				0.213 ^2^
Indoor kitchen in specific room	20 (55.6%)	16 (44.4%)	36	
Indoor kitchen no specific room	85 (52.1%)	78 (47.9%)	163	
Outdoor kitchen in specific room	13 (43.3%)	17 (56.7%)	30	
Outdoor kitchen no specific room	40 (40.4%)	59 (59.6%)	99	
**Child has a personal plate**				0.285 ^3^
Yes	18 (39.1%)	28 (60.9%)	46	
No	141 (49.8%)	142 (50.2%)	283	
**Sewage disposal**				0.572 ^2^
Adapted	132 (47.7%)	145 (52.3%)	277	
Unsuitable	27 (51.9%)	25 (48.1%)	52	
**Household waste disposal**				1.000 ^3^
Adapted	127 (48.3%)	136 (51.7%)	263	
Unsuitable	27 (48.2%)	29 (51.8%)	56	
Unknown	5 (50.0%)	5 (50.0%)	10	
**Guardian’s hand washing**				1.000 ^3^
No hand washing	2 (40.0%)	3 (60.0%)	5	
Only with cold and/or hot water	92 (48.7%)	97 (51.3%)	189	
With water and soap	64 (48.5%)	68 (51.5%)	132	
**Number of handwashing of the guardian per day**				0.770 ^2^
1–2 times	12 (42.9%)	16 (57.1%)	28	
3–4 times	73 (50.0%)	73 (50.0%)	146	
≥5 times	73 (47.7%)	80 (52.3%)	153	
**Type of kitchen**				0.213 ^2^
Indoor kitchen in specific room	20 (55.6%)	16 (44.4%)	36	
Indoor kitchen no specific room	85 (52.1%)	78 (47.9%)	163	
Outdoor kitchen in specific room	13 (43.3%)	17 (56.7%)	30	
Outdoor kitchen no specific room	40 (40.4%)	59 (59.6%)	99	
**Child has a personal plate**				0.285 ^3^
Yes	18 (39.1%)	28 (60.9%)	46	
No	141 (49.8%)	142 (50.2%)	283	
**Sewage disposal**				0.572 ^2^
Adapted	132 (47.7%)	145 (52.3%)	277	
Unsuitable	27 (51.9%)	25 (48.1%)	52	
**Household waste disposal**				1.000 ^3^
Adapted	127 (48.3%)	136 (51.7%)	263	
Unsuitable	27 (48.2%)	29 (51.8%)	56	
Unknown	5 (50.0%)	5 (50.0%)	10	

^1^ Chi-square test. ^2^ Wilcox test. ^3^ T-Student test. ^4^ Fisher exact test. IQR = Interquartile range. Missing data: Weight at birth (n = 175), history fever (n = 2) and rotavirus vaccination (n = 2).

**Table 3:** Parasitic carriage of the study population according to virus positivity (N = 329).

	Viral Detection		Total (N = 329)	*p*-Value
	No (N = 159)	Yes (N = 170)		
	n (%)	n (%)	n (%)	
Presence of at least one parasite				0.370 ^1^
Yes	124 (50.0%)	124 (50.0%)	248 (75.4%)	
No	34 (44.2%)	43 (55.8%)	77 (23.4%)	
* Giardia intestinalis *				0.873 ^1^
Yes	40 (49.4%)	41 (50.6%)	81 (24.6%)	
No	118 (48.4%)	126 (51.6%)	244 (74.2%)	
* Ascaris lumbricoides *				0.013 ^1^
Yes	90 (55.6%)	72 (44.4%)	162 (49.2%)	
No	68 (41.7%)	95 (58.3%)	163 (49.5%)	
* Trichuris trichiura *				0.637 ^1^
Yes	103 (47.7%)	113 (52.3%)	216 (65.6%)	
No	55 (50.5%)	54 (49.5%)	109 (33.1%)	
* Enterobius vermicularis *				0.614 ^2^
Yes	2 (66.7%)	1 (33.3%)	3 (0.9%)	
No	156 (48.4%)	166 (51.6%)	322 (97.9%)	

^1^ Chi-square test. ^2^ Fisher exact test. Missing data: Presence of at least one parasite (n = 4), *G. intestinalis* (n = 4), *A. lumbricoides* (n = 4), *T. trichiura* (n = 4) and *E. vermicularis* (n = 4).

**Table 4:** Risk factor estimation using multivariate logistic regression model of viral detection.

	Viral Detection		Crude OR (CI 95%) *	Adjusted OR ** (CI 95%) *	*p*-Value
	No (N = 149)	Yes (N = 155)			
**Child’s food diversity score**					0.03
Unsuitable	49 (32.9%)	70 (45.2%)	ref.	ref.	
Adequate	100 (67.1%)	85 (54.8%)	0.59 (0.37–0.95)	0.58 (0.35–0.94)	
**Nutritional status of the mother**					
Normal	102 (68.5%)	86 (55.5%)	ref.	ref.	
Undernourished	23 (15.4%)	20 (12.9%)	1.03 (0.53–2)	1.07 (0.54–2.11)	0.791
Overnourished	24 (16.1%)	49 (31.6%)	2.42 (1.37–4.27)	2.45 (1.38–4.42)	0.003
***A. lumbricoides* carriage**					0.021
Yes	87 (58.4%)	69 (44.5%)	ref.	ref.	
No	62 (41.6%)	86 (55.5%)	1.75 (1.11,2.75)	1.75 (1.09–2.83)	

* CI: Confidence Interval of 95%. ** Adjusted with age, gender, and season of inclusion.

## Data Availability

All data generated or analyzed during this study are included in the published article and its supplementary information files.

## Supplementary Materials

The following supporting information can be downloaded at: https://www.mdpi.com/article/10.3390/pathogens12081009/s1, Supplementary Table S1: List of primers and probes; Supplementary Table S2: Association of the growth status of children with adenovirus type F40/41 infection; Supplementary Table S3. Correlation of adenovirus non-type F40/41 carriage with clinical symptoms observed in the study population (N = 251); Supplementary Table S4. Sensitivity and specificity of the final model; Supplementary Table S5. Bivariate analysis of risk factors statistically associated with viral carriage (*p*-value < 0.20).
