# Supplementary material for: Factors Associated with Carriage of Enteropathogenic and Non-Enteropathogenic Viruses: A Reanalysis of Matched Case-Control Data from the AFRIBIOTA Site in Antananarivo, Madagascar
Source: Pathogens. 2023 Aug 2;12(8):1009. doi: 10.3390/pathogens12081009 (PMC10459613; doi:10.3390/pathogens12081009)
Supplement: Supplementary file 1 [file pathogens-12-01009-s001.zip › pathogens-2481295-supplementary.pdf]

Supplementary materials

Supplementary Table S1. List of primers and probes

**Targeted viruses**	**Forward primer**	**Reverse primer**	**Probe**	**Fluorophore[1]**	**Target gene / Access number-GenBank**	**Reference**
Adenovirus-pan	GCCACGGTGGGGTTTCTAAACTT	GCCCCAGTGGTCTTACATGCACATC	TGCACCAGACCCGGGCTCAGGTACTCCGA	FAM-BHQ1	Hexon/ J01917	Elfving K, 2014
Adenovirus 40/41[2]	TGCCCGCGCCACCGAT	GAGCCACAGTGGGGTTTCTG	CCAGGCTGAAGTACG	FAM-BHQ1	Hexon/ X51782	Elfving K, 2014
Rotavirus	(1) AACCATCTACACATGACCCTCTATGA<br>(2) AACCATCTTCACGTAACCCTCTATGA	GGTCACATAACGCCCCTATAGC	CAATAGTTAAAAGCTAACACTGTCAAA	FAM-TAMRA	Nonstructural protein 3 (NSP3) / X81436	Elfving K, 2014
Astrovirus	GACTGCWAAGCAGCTTCGTGA	GCTAGCCATCACACTTCTTTGGTCCT	TCACAGAAGAGCAACTCCATCGCATTTG	Cy5-BHQ1	Pol-capsid junction/ NC_001943	Elfving K, 2014
Norovirus GII	TGGAYTTTTAYGTGCCCAG	CGACGCCATCTTCATTCAC	AGCCAGATTGCGATCGCCC	VIC-BHQ1	Pol-capsid junction/ X86557	Elfving K, 2014

[1]probe labeled with fluorophores: FAM, 6-carboxyfluorescein; VIC, 4,7,2=-trichloro-7=-phenyl-6-carboxyfluorescein; Cy5, 1,1′-dimethyl-3,3,3′,3′-tetramethylindocarbocyanine; TAMRA, 6-carboxytetramethylrhodamine; BHQ1, Black Hole Quencher 1.

[2] Run separately as a complementary analysis.

Supplementary Table S2. Association of the growth status of children with adenovirus type F40/41 infection.

	**NS or controls** (N=111)	**MS** (N=47)	**SS** (N=41)	**Overall** (N=199)	***p-value****
Adenovirus type40/41					0.72
No	96 (86.5%)	43 (91.5%)	37 (90.2%)	176 (88.4%)	
Yes	15 (13.5%)	4 (8.5%)	4 (9.8%)	23 (11.6%)	

NS or controls: Non-stunted; MS: moderately stunted; SS: severely stunted; N: total number of samples analyzed; Comparison between groups (controls and stunted children MS+SS) were determined using Fisher’s exact test according to their conditions of use. *A p-value < 0.05 was considered statistically significant

Supplementary materials

Supplementary Table S3. Correlation of adenovirus non-type F40/41 carriage with clinical symptoms observed in the study population (N=251).

	Adenovirus detection			
	**No (N=159)**	**Yes (N=92)**	**Total (N=251)**	***p*-value**
	**n (%)/median [IQR]**	**n (%)/median [IQR]**	**n/median [IQR]**	
**Age**				0.540[1]
Median	44.3 [34.8. 53.1]	43.4 [32.8. 52.3]	43.2 [33.6. 52.9]	
**Growth status**				
Non-stunted	52 (38.8)	82 (61.2)	134	0.466[2]
Moderately stunted	19 (30.1)	44 (69.8)	63	
Severely stunted	21 (38.9)	33 (61.1)	54	
**Stuffy nose**				
Yes	84 (68.3)	39 (31.7)	123	0.111[2]
No	75 (58.6)	53 (41.4)	128	
**Rhinorrhea**				
Yes	105 (67.7)	50 (32.2)	155	0.066[2]
No	54 (56.3)	42 (43.7)	96	
**Cough**				
Yes	63 (70.0)	27 (30.0)	90	0.102[2]
No	96 (59.6)	65 (40.4)	161	

[1] Chi square-test. [2] Wilcoxon-Mann-Whitney test.

Supplementary materials

Supplementary Table S4. Sensitivity and specificity of the final model.

Predicted	Actual	
	**No**	**Yes**
**No**	113	77
**Yes**	36	78

Sensitivity of the model: 50.3%, IC 95% (42.2 - 58.4); Specificity of the model: 75.8%, IC 95% (68.2 - 82.5)

Supplementary Table S5. Bivariate analysis of risk factors statistically associated with viral carriage (p-value < 0.20).

	Food score diversity of the child	Nutritional status of the mother	Weight at birth	Age	Sex	Respiratory infection history	Drinking water treatment	Ascaris	Child eats in the plate of the guardian	Mother's schooling level	Household waste disposal
Food score diversity of the child	1	0.705	0.4097	0.435	0.056	1.000	< 0.001	0.401	0.991	0.003	0.759
Nutritional status of the mother		1	0.07524	0.9921	0.257	0.447	0.473	0.412	0.151	0.143	0.726
Weight at birth			1	0.9219	0.9614	0.6348	0.5491	0.9444	0.6793	0.9968	0.7794
Age				1	0.84	0.2986	0.9389	0.07076	0.4505	0.4265	0.9073
Sex					1	1.000	0.211	0.291	0.739	0.305	0.682
Respiratory infection history						1	1.000	1.000	0.220	0.567	0.286
Drinking water treatment							1	0.735	0.102	0.151	0.203
Ascaris								1	0.081	0.017	0.354
Child eat in the plate of the guardian									1	0.316	0.509
Mother's schooling level										1	0.683
Household waste disposal											1
